# Interdisciplinary approaches based on imaging radar enable cutting-edge cultural heritage applications

**DOI:** 10.1093/nsr/nwab123

**Published:** 2021-07-07

**Authors:** Fulong Chen, Huadong Guo, Deodato Tapete, Nicola Masini, Francesca Cigna, Rosa Lasaponara, Salvatore Piro, Hui Lin, Peifeng Ma

**Affiliations:** Key Laboratory of Digital Earth Science, Aerospace Information Research Institute, Chinese Academy of Sciences, China; International Centre on Space Technologies for Natural and Cultural Heritage, China; Key Laboratory of Digital Earth Science, Aerospace Information Research Institute, Chinese Academy of Sciences, China; International Centre on Space Technologies for Natural and Cultural Heritage, China; Italian Space Agency, Italy; Institute of Science of Cultural Heritage, National Research Council, Italy; Institute of Atmospheric Sciences and Climate, National Research Council, Italy; Institute of Methodologies for Environmental Analysis, National Research Council, Italy; Institute of Science of Cultural Heritage, National Research Council, Italy; School of Geography and Environment, Jiangxi Normal University, China; Institute of Space and Earth Information Science, The Chinese University of Hong Kong, China

## Abstract

By analysing the technical advantages and characteristics of imaging radar in cultural heritage, we provide new insights for the future development of cutting-edge Digital Heritage approaches through technical integration and interdisciplinary synergy.

Heritage assets reflecting memories of the past are increasingly sustained by societies as a necessary condition for the continuing evolution of people's culture. The call for ‘strengthening efforts to protect and safeguard the world's cultural and natural heritage’ in United Nations Sustainable Development Goal (SDG) 11 [1] is a clear acknowledgement of the role of heritage in sustaining resilient societies and lifestyles.

To understand and safeguard irreplaceable cultural assets, archaeological prospection and heritage preservation are two interconnected and essential research domains. Prospection aims at archaeological discovery and documentation, to bring back to light buried and hitherto unknown structures, antiquities and even extensive paleo-landscapes. Preservation orients us to prevent further deterioration and to design conservation measures to enhance heritage sustainability by monitoring the condition of heritage assets through time. Both archaeological investigation and heritage preservation include the use of all possible invasive and non-invasive means.

Over the last few decades, an increasing number of technologies have been applied to facilitate sustainability goals of cultural heritage [2,3], and among them remote sensing is recognized as a viable tool [4] owing to its capacity for non-invasive, multi-scale observations and measurements over large territories. Optical methods have been widely used because the data are intuitive and usually easy to interpret. As a complementary tool, imaging radar has emerged as an effective means for systematic and continuous long-term remote observations of cultural heritage to improve documentation, monitoring, preservation and management.

Imaging radar includes airborne/spaceborne Synthetic Aperture Radar (SAR), Ground Penetrating Radar (GPR) and Ground-Based SAR (GB-SAR), whose operation characteristics are generally diverse but complementary, depending on their peculiar observation capabilities coupled with investigation needs and scales (Fig. 1). Following a holistic approach, the usage of these different instrumentations should be conceived according to a multi-sensor/data and multi-scale perspective, although constraints due to data and instrumentation accessibility (e.g. lack of funds) may prevail in some circumstances.

**Figure 1.**
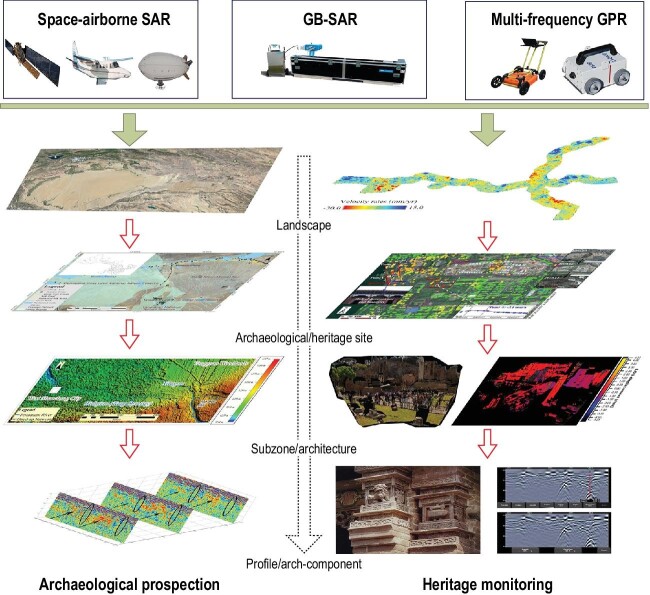
Framework of multi-platform imaging radar in cultural heritage applications with complementary observation views and monitoring scopes.

Across large landscapes, satellite SAR imagery is capable of revealing ancient drainage patterns [5], palaeo-channels and other human traces. Confidence in the observed archaeological traces improves as imaging resolution from satellite or aircraft increases. Then archaeological remains can be further detailed using geo-archaeology tools and close-range remote sensing methods, including GPR (in use for over three decades) to explore anthropogenic layers and determine their depths [6]. Hence, such a multi-scale SAR-GPR integrated approach could be effective for archaeological site detection across palaeo-landscapes (Fig. 1). In this domain, SAR investigations proved successful, especially for analysing cause-effect mechanisms between backscattering signals and archaeological proxy indicators, linked to micro-topographical variations and changes in moisture content.

The benefits brought by technology integration are particularly relevant in the desert and semi-arid regions, where optical remote sensing is limited, whereas SAR can offer large-scale imaging, subsurface penetration and effective feature discrimination. Moreover, today a huge amount of satellite SAR data can be promptly processed using cloud tools (also available as open-access tools such as Google Earth Engine) to automatically identify archaeological traces, using artificial intelligence as machine and deep learning approaches [7]. After that, in-depth archaeological investigations need to be implemented at the site level, or even in specific subzones of archaeological interest, using high-resolution SAR products and GPR subsurface imaging. The performance of GPR prospection in a dry-arid environment is maximized when soil moisture is low and the penetration of radar signatures is enhanced.

Moreover, being sensitive to subtle motions, spaceborne Multitemporal SAR Interferometry (MTInSAR) is recommended for monitoring purposes. MTInSAR detects millimetric deformation anomalies at the cultural-monument scale [8], providing early-warning signals for identifying zones of structural instability at individual monuments which could then be monitored using the 2D-static (motion) measurements of GB-SAR [9]. Details on issues affecting monument components can be further clarified using the health diagnosis model of portable GPR, which helps identify structural fissures or cavities [10]. The integrated use of MTInSAR–GB-SAR–GPR does improve the resolution and provide multi-scale/level information ranging from the identification of cracks and deformations of masonry structures and artefacts, to the detection of fresco detachment or the identification of the diverse construction phases/techniques of monuments and works of art (Fig. 1).

A dedicated literature review based on the Web of Science database reveals a clear but uneven increase in the use of imaging radar technology in cultural heritage management over the last 30 years; from the first publication in 1992 to ∼40 papers/yr between 2015 and 2020, with >80% publications focusing on GPR-based case studies. Surprisingly, despite the intrinsic complementarities, the integrated use of GPR, GB-SAR and airborne-satellite SAR is rare. It is also revealed that collaboration had become a necessary condition for interdisciplinary heritage studies, particularly in complex landscapes and situational contexts that call for knowledge and expertise from scientists, engineers and other stakeholders. Institution-level cooperation has become a mainstream trend in scientific and industrial communities in order to achieve benefits through in-depth theoretical investigations and technical applications.

A viable pathway could be the design of pilot projects enhancing trans- and interdisciplinary collaborations at the international level, as already experienced in the past in the framework of the United Nations Educational, Scientific and Cultural Organization (UNESCO) and European Space Agency (ESA) Open Initiative, which focused on ‘the Use of Space Technologies to Support the World Heritage Convention’.

Launch and enlargement of such initiatives would be welcome, to provide an optimum platform on a worldwide scale for building case studies on digital heritage applications using integrated imaging technologies (with particular emphasis on radar). A partnership with international organizations, including UNESCO’s Category 1 and 2 centres and institutes, is proposed. It contributes to the execution of UNESCO’s programme and addresses the gap of Tier III SDG indicators (i.e. 11.4.1) by way of the International Centre on Space Technologies for Natural and Cultural Heritage (HIST), whose mission is the protection and safeguarding of cultural and natural heritage.
